# Regulation of CD45 phosphatase by oncogenic ALK in anaplastic large cell lymphoma

**DOI:** 10.3389/fonc.2022.1085672

**Published:** 2023-01-09

**Authors:** Giulia Mura, Elif Karaca Atabay, Matteo Menotti, Cinzia Martinengo, Chiara Ambrogio, Gloria Giacomello, Maddalena Arigoni, Martina Olivero, Raffaele A. Calogero, Roberto Chiarle, Claudia Voena

**Affiliations:** ^1^ Department of Molecular Biotechnology and Health Sciences, University of Torino, Torino, Italy; ^2^ Department of Pathology, Children’s Hospital and Harvard Medical School, Boston, MA, United States; ^3^ Molecular Biotechnology Center (MBC), University of Torino, Torino, Italy; ^4^ Candiolo Cancer Institute, FPO-IRCCS, Candiolo, Torino, Italy; ^5^ Department of Oncology, University of Torino, Torino, Italy

**Keywords:** anaplastic large cell lymphoma, ALK, CD45, phosphatases, resistance, tyrosine kinase inhibitor

## Abstract

Anaplastic Large Cell Lymphoma (ALCL) is a subtype of non-Hodgkin lymphoma frequently driven by the chimeric tyrosine kinase NPM-ALK, generated by the t (2,5)(p23;q35) translocation. While ALK+ ALCL belongs to mature T cell lymphomas, loss of T cell identity is observed in the majority of ALCL secondary to a transcriptional and epigenetic repressive program induced by oncogenic NPM-ALK. While inhibiting the expression of T cell molecules, NPM-ALK activates surrogate TCR signaling by directly inducing pathways downstream the TCR. CD45 is a tyrosine phosphatase that plays a central role in T cell activation by controlling the TCR signaling and regulating the cytokine responses through the JAK/STAT pathway and exists in different isoforms depending on the stage of T-cell maturation, activation and differentiation. ALK+ ALCL cells mainly express the isoform CD45RO in keeping with their mature/memory T cell phenotype. Because of its regulatory effect on the JAK/STAT pathway that is essential for ALK+ ALCL, we investigated whether CD45 expression was affected by oncogenic ALK. We found that most ALK+ ALCL cell lines express the CD45RO isoform with modest CD45RA expression and that NPM-ALK regulated the expression of these CD45 isoforms. Regulation of CD45 expression was dependent on ALK kinase activity as CD45RO expression was increased when NPM-ALK kinase activity was inhibited by treatment with ALK tyrosine kinase inhibitors (TKIs). Silencing ALK expression through shRNA or degradation of ALK by the PROTAC TL13-112 caused upregulation of CD45RO both at mRNA and protein levels with minimal changes on CD45RA, overall indicating that oncogenic ALK downregulates the expression of CD45. CD45 repression was mediated by STAT3 as demonstrated by ChIP-seq data on ALCL cells treated with the ALK-TKI crizotinib or cells treated with a STAT3 degrader. Next, we found that knocking-out CD45 with the CRISPR/Cas9 system resulted in increased resistance to ALK TKI treatment and CD45 was down-regulated in ALCL cells that developed resistance *in vitro* to ALK TKIs. Overall, these data suggest that CD45 expression is regulated by ALK *via* STAT3 and acts as a rheostat of ALK oncogenic signaling and resistance to TKI treatment in ALCL.

## Introduction

Anaplastic large cell lymphoma (ALCL) is a subtype of T cell lymphoma characterized by a mature T cell phenotype and by the expression of CD30, a member of the tumor necrosis factor (TNF) receptor superfamily (1, 2). The majority of ALCL harbor the recurrent t (2,5)(p23;q35) translocation that generates the oncogenic fusion protein NPM-ALK. In ALK+ ALCL, oncogenic NPM-ALK sustains the activated phenotype of neoplastic T cells through several pathways, including the JAK/STAT3, PI3K/AKT and MAPK pathways (3). Despite displaying an activated T cell phenotype and TCR rearrangements, ALK+ ALCL shows the variable loss of T-cell markers (4). In ALK+ ALCL oncogenic ALK can modulate T cell identity by inducing a transcriptional and epigenetic repressive program that causes a loss of expression of critical TCR downstream molecules, such as CD3ε, ZAP70, LAT and SLP76. Accordingly, oncogenic ALK can bypass TCR activation and independently activate downstream signaling mediators of the TCR signaling, as we and others previously demonstrated (5, 6). Activation of the TCR signaling in T cell depends on the modulation of several molecules, such as accessory molecules (CD4 or CD8), tyrosine kinases and tyrosine phosphatases. The transmembrane tyrosine phosphatase (PTPase) CD45, encoded by the *PTPRC* gene, is a crucial regulator of proper TCR signaling and is required for T-cell function (7–9). Depending on the stage of T-cell maturation, activation and differentiation, CD45 exists in different isoforms due to alternative splicing (10, 11). Therefore, CD45 has become a diagnostic marker to distinguish between different T cells because naïve T cells express the isoform CD45RA, while memory and activated T cells express CD45RO (12). Besides TCR regulation through the Src-family kinase Lck, CD45 is a regulator of cytokine activation through the negative regulation of the JAK/STAT pathway (12–14). Indeed, CD45 directly dephosphorylates all the JAK family members resulting in decreased JAK activity and, consequently, affecting STAT transcription factors (15). Accordingly, CD45 inactivating mutations in some T acute lymphoblastic leukemia (T-ALL) were associated with increased JAK/STAT signaling. Down-regulation of CD45 in T-ALL cell lines harboring JAK1 mutation caused increased proliferation and sensitized cells to the JAK inhibitor ruxolitinib (16, 17). In ALK+ ALCL, the JAK/STAT pathway is a critical downstream mediator of ALK oncogenic signaling sustaining the neoplastic phenotype and contributing to the survival of lymphoma cells (18, 19); therefore, we investigated whether oncogenic ALK affected CD45 expression and activity. Here, we show that in ALK+ ALCL NPM-ALK downregulates CD45 expression both at transcriptional and protein levels. We demonstrate that NPM-ALK inhibition by ALK specific tyrosine kinase inhibitors (TKIs) or degradation by the newly developed PROTAC TL13-112 induced the upregulation of CD45, specifically the CD45RO isoform that is mainly expressed in ALK+ ALCL cell lines. CD45 repression was mediated by STAT3, as demonstrated by ChIP-seq data on ALCL cells treated with ALK TKIs and confirmed by treatment with the STAT3 degrader SD36. In addition, by RNA-sequencing analysis of crizotinib-resistant ALCL cells we found that CD45 mRNA was significantly downregulated in the crizotinib-resistant cells compared to the sensitive counterpart. In accordance, knocking out CD45 in ALK+ ALCL cells by the CRISPR/Cas9 system induced crizotinib resistance. In conclusion, our data suggest that NPM-ALK modulates CD45 expression through STAT3 activity and that CD45 loss can induce resistance to ALK TKIs.

## Materials and methods

### Cell lines and reagents

Human ALK+ ALCL cell lines were maintained in RPMI 1640 medium supplemented with 10% fetal bovine serum (FBS), 2% penicillin and streptomycin and 1% glutamine. Cell lines were grown at 37°C in a humidified atmosphere with 5% CO2. ALK+ cell lines (TS, SU-DHL1, JB6, KARPAS-299) and ALK- cell lines (CEM, JURKAT and NAMALWA) were obtained from DSMZ cell bank (German Collection of Microorganisms and Cell Cultures). ALK+ ALCL cell line, COST was kindly provided by Dr. Laurence Lamant (Institut Universitaire du Cancer Toulouse Oncopole, Toulouse, France). All cell lines were routinely tested for Mycoplasma contamination *via* PCR and always tested negative

ALK tyrosine kinase inhibitor, crizotinib, was obtained from Pfizer (New York, NY, USA) and dissolved in DMSO for *in vitro* experiments. ALK degrader TL13-112 (cat# 6745/5) was purchased from Tocris Bioscience and STAT-3 degrader SD36 (cat# HY-129602) was purchased by MedChemExpress. They were dissolved according to manufacturer’s instructions.

Inducible ALK short hairpin (shRNA) SU-DHL-1 and TS (SU-DHL-1 TTA A5 and TS TTA A5, respectively) cells were obtained by co-transduction with pLV-tTRKRAB (TTA) vector and pLVTHM vector containing the H1 promoter ALK-shRNA (A5) cassette. These cells undergo NPM-ALK silencing when 1 μg/mL doxycycline is added to the medium as previously described (19).

### Generation of crizotinb-resistant cells

Crizotinib-resistant ALK+ ALCL (CR) cell lines were generated in the laboratory by culturing cell lines with progressively increasing concentration of crizotinib (10 nM – 1000 nM) over time. After 2-3 passages at the same crizotinib concentration, a higher dose of crizotinib was added to the medium until a final concentration of 1000 nM was achieved.

### Virus preparation and cell transduction

Lentiviruses were produced using the 3rd generation production system. Briefly, HEK293T cell lines were cultured in DMEM with 10% FBS. Cells at 70% confluency were co-transfected with pVSVG, pCMVR8.74, pRSV-Rev and a lentiviral vector expressing the construct of interest. Media was replenished 12/18h after transfection, and the supernatant was collected after 24 and 48h. Collected supernatants were filtered through 0.22 μm filter, concentrated by ultracentrifugation (50,000 X g for 2hr) and resuspended in 500 μl of sterile PBS. For infection, 5 × 10^4^ cells were infected with the prepared lentivirus along with polybrene (8μg/ml), cells with viral particles were spun down at 2400 RPM for 90 minutes and then incubated at 37° overnight. The infectivity was determined after 72 hours by FACS analysis of GFP-positive cells.

### CD45 KO by CRISPR/Cas9 gene editing

CD45-KO ALCL cell lines were generated using CRISPR/Cas9-mediated gene targeting. Single-guide RNAs (sgRNAs) targeting exon 3 of PTPRC (5’- TAATTCTTACCAGTGGGGGA-3’) or exon 7 (5’- GGTAATATCACCTATTGTTG-3’) were designed using the using the Deskgen tool and ligated into the Lenti-CRISPR v2 vector. Lentiviral particles were produced in HEK293T cells and viral supernatant used to transduce ALCL cell lines (TS and JB6), according to the protocol described above. CRISPR/Cas9 empty vector lentivirus was used as control. Puromycin was added after 48 hours, using the minimal toxic dose per cell line to select transduced cells. The selected cells were then analyzed by Flow cytometry and Western blot to check CD45 KO. CD45 KO TS and JB6 cells were grown in crizotinib for 14. The crizotinib-resistant population was used for viability assay and western blot analysis.

### Cell lysis and Western blot analysis

Whole cell extracts were prepared by resuspending the pelleted cells in GST-FISH lysis buffer containing (10nM MgCl2, 150nM NaCl, 1% NP-40, 2% glycerol, 1mM EDTA, 25nM HEPES pH 7.5), mixed with 1mM phenylmethylsulphonyl fluoride (PMSF), 10mM sodium fluoride, 1mM Na3VO4, a protease inhibitor cocktail (PIC) and a phosphatase inhibitors cocktail incubated on ice for 20-30 minutes. Cell lysates were collected by centrifugation at 12,000 rpm for 10 minutes at 4°C. Supernatants were analyzed for protein concentration with a Bio-Rad DC protein assay kit (Bio-Rad Laboratories) and stored at −80°C. The following primary antibodies were used in the study: anti-CD45 (1:1000; Cell Signaling Technology), anti-CD45RO (1:1000; Cell Signaling Technology), anti-ALK (D5F3) (1:1000; Cell Signaling Technology), anti-phospho-ALK (Y1604) (1:1000 Cell Signaling Technology), anti-β-tubulin (1:2000 Sigma), anti-β-actin (1:1000; Cell Signaling Technology), anti-STAT3 (79D7) (1:1000; Cell Signaling Technology), anti-phospho-STAT3 (Tyr705) (1:1000; Cell Signaling Technology), anti-pospho-ERK1/2 (Thr202/Tyr204) (1:1000; Cell Signaling Technology), anti-ERK1/2 (1:1000; Cell Signaling Technology).

### Cell viability assay

Viability was assessed with the CellTiter-Glo 2.0 Assay (Promega) as indicated by manufacturer. Cells (5000 cells/well) were plated in triplicates in 96-well plates and treated with an interval of crizotinib concentrations for 72 hours. Chemiluminescence was red using the GloMax-Multi Detection System (Promega). Data were analyzed with GraphPad Prism (Version 8.4.2).

### RNA sequencing on crizotinib-sensitive and -resistant ALCL cell lines

*Total RNA purification*. RNA was purified from wild type and crizotinib resistant cell lines using RNeasy mini kit (Qiagen). Total RNA was estimated quantitatively and qualitatively with Qubit RNA BR kit (ThermoFisher Scientific) and Agilent 2100 Bioanalyzer (Agilent Technologies) and then stored at -80°C until use. 1 μg of RNA was treated with DNAse (DNA-free™ kit, Ambion) and used for RNA-sequencing.

*RNA-sequencing*. Coding transcriptome was sequenced from libraries generated using TruSeq RNA Access library prep kit (Illumina) following manufacturer’s instructions and starting from 40 ng of DNA-free RNA from wild type and crizotinib resistant cells. Libraries were quantified with Qubit DNA ds HS (LifeTechnologies) and run at the concentration of 1.6 pM on the NextSeq500 Illumina sequencer in 75 nts paired end sequencing mode following manufacturer instruction.

*Bioinformatics analysis*. Analyses were performed using the tools embedded in docker4seq package [https://pubmed.ncbi.nlm.nih.gov/29069297/], to guarantee bioinformatically reproducible results [https://pubmed.ncbi.nlm.nih.gov/30367595/]. Coding transcriptome RNAseq quantification was done using STAR/RSEM (20, 21). Reads were mapped against human genome (hg38) using ENSEMBL GTF (version 99) annotation. Differential expression analysis was done using DESeq2 Bioconductor package (22). RNAs with a |Log_2_FC| ≥ 1 and an adjusted p-value ≤ 0.1 were considered as differentially expressed.

### Accession numbers

The RNA-seq generated from of ALCL cell lines sensitive and resistant to crizotinb are available at NCBI Gene Expression Omnibus (GEO) under accession number GSE173306 with token ohqdkaawnfkpbon.

### Quantitative real-time PCR

Total RNA was extracted from pelleted cells using TRIZOL solution (Euroclone), followed by cDNA preparation from 1µg. cDNA was transcribed using LunaScript^®^ RT SuperMix Kit. cDNA products were quantified by real-time PCR using SYBR Green Supermix (Biorad) on CFX Opus Real-Time PCR System (Bio-Rad). The reactions were performed in triplicate. Housekeeping human acidic ribosomal protein (HuPO) was used for normalization, according to the formula 2-ΔΔCt, where the ΔCt=¼ Ct (threshold cycle) gene of interest -Ct internal control, as indicated by the manufacturer.

Primers to detect total CD45 and the RA and RO isoforms were designed using Primer Blast. Primers for total CD45 were designed at exons 7 and 8; for the RA isoform at exons 3 and 4; for the RO isoform the forward primer was designed at the splicing site of 3/7 exons and the reverse primer at exon 7. Primers are indicated in the following table:

**Table:** 

Gene	Forward primer	Reverse primer
CD45 (total)	5’-CTTCTGGAAGCGCTGTCATT-3’	5’-ATGCACCTCATTGTTTGTGC-3’
CD45RA	F 5’-CCCCGGACTCTTTGGATAAT-3’	5’-AGGGTTGAGTTTTGCATTGG-3’
CD45RO	5’-CCCCACTGATGCCTACCTTA-3’	5’-TCACATGTTGGCTTAGATGGAG-3’
Hupo	5’-GCTTCCTGGAGGGTGTCC-3’	5’-GCTTCCTGGAGGGTGTCC-3’

### Flow cytometry analysis

For each cell line, 1 x 10^5^ cells were resuspended in PBS and stained with the following antibodies: CD45 PerCP anti-human (Clone: HI30, BioLegend), CD45RA PerCP Anti-human (Clone: REA1047 Miltenyi Biotec), CD45RO PE Anti-human (Thermo Fisher Scientific). Staining was performed for 20 minutes at Room Temperature (RT) in the dark. Next, cells were washed with PBS and events were acquired using the BD FACSCelesta™ flow cytometer (BD Biosciences, USA). Data were analyzed with Flow Jo v10. Software.

### Statistical analysis

Values were presented as mean ± standard deviation (SD). Statistical significance (p) was calculated using the Student’s t test (GraphPad Software, La Jolla, CA, USA). Each experiment was independently performed at least two times and a p-value of <0.05 was considered statistically significant, where *= p<0.05, **= p<0.01, ***= p<0.001 and ****= p<0.0001.

## Results

### Oncogenic NPM-ALK controls CD45 expression in ALK+ ALCL

**Figure 1:**
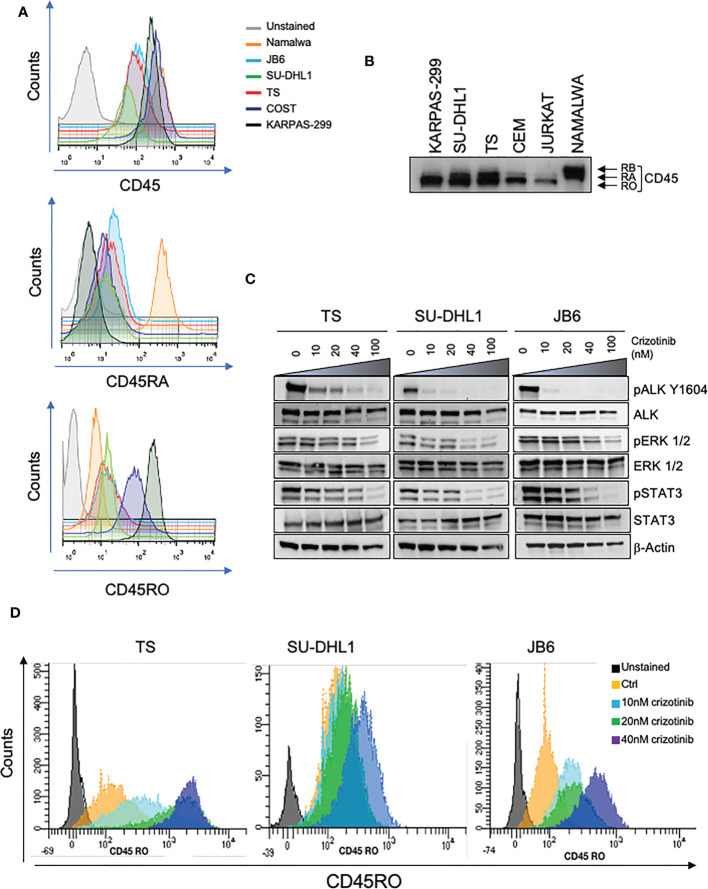
CD45 is expressed in ALK+ ALCL cells. **(A)** CD45, CD45RA, CD45RO basal cell surface expression in ALK + ALCL cells (TS, SU-DHL1, JB6, COST and KARPAS-299) and in Burkitt Lymphoma cells (NAMALWA) measured by flow-cytometry. A representative experiment out of three is shown. **(B)** Immunoblot analysis performed on human ALK+ ALCL cells lines and Burkitt lymphoma cells (NAMALWA), Lymphoblastic cells (CEM), T acute lymphoblastic leukemia cells (JURKAT) blotted with the indicated antibodies. A representative experiment out of two is shown. **(C)** Immunoblot analysis on three representative ALK+ ALCL human cell lines (TS, JB6 and SU-DHL1) treated with increasing concentrations of ALK inhibitor crizotinib for 3 hours. Cell lysates were blotted with the indicated antibodies. **(D)** CD45RO cell surface expression intensity measured by flow-cytometry in ALK+ ALCL cell lines treated with the indicated concentrations of crizotinib for 96 hours. The experiment and Western blot were performed one time on three independent cell lines with similar results.

The transmembrane tyrosine PTPase CD45 is a key regulator of antigen receptor signaling in T and B cells that express high levels of CD45 at all maturation stages (23). In addition, CD45 plays an inhibitor role in the JAK/STAT pathway in response to cytokine signaling in hematopoietic cells (15). Since in ALK+ ALCL, the JAK/STAT pathway is a crucial signaling mediator activated by oncogenic NPM-ALK, we investigated whether NPM-ALK regulated CD45 expression. First, we assessed the levels of expression of CD45, and specifically which isoform was mainly expressed in ALK+ ALCL since CD45 is expressed on the cell surface in different isoforms depending on the stage of T cell maturation, activation and differentiation (9). By flow-cytometry analysis, we found that ALK+ ALCL cell lines expressed variable levels of CD45, mainly the CD45RO isoform accordingly with the observation that primary ALK+ ALCL show a mature/memory T cell phenotype (**Figure 1A**) (4, 24). In contrast, the CD45RA isoform was expressed at lower levels than NAMALWA, a control B cell lymphoma cell line. Western blot analysis also confirmed that ALK+ ALCL cell lines preferentially expressed the CD45RO isoform (**Figure 1B**). Altogether, we demonstrated that ALK+ ALCL mostly showed a CD45RO^high^/CD45RA^low^ phenotype.

**Figure 2:**
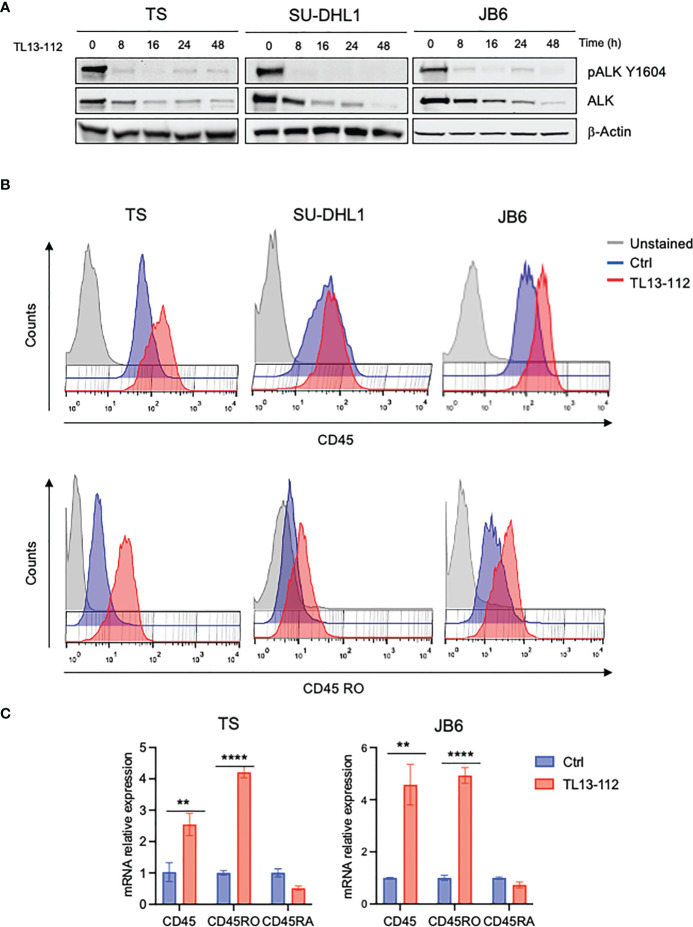
CD45 and CD45RO are derepressed by ALK degradation. **(A)** Immunoblot analysis of ALK+ ALCL cells (TS, SU-DHL1 and JB6) treated with ALK degrader TL13-112 (25, 10and 50nM, respectively) for the indicated time-points. Total cell lysates were blotted with the indicated antibodies. β-actin was used as a loading control. A representative Western blot out of two is shown for each cell line. **(B)** CD45 and CD45RO cell surface expression intensity measured by flow-cytometry in ALK+ ALCL cell lines treated with ALK degrader TL13-112. ALCL cells were treated with the same concentrations as in **(A)** for 24h (JB6) and 48h (TS and SU-DHL1). A representative experiment out of two is shown for each cell line. **(C)** qRT-PCR analysis of CD45, CD45RO, CD45RA mRNA expression performed on TS and JB6 ALCL cell lines treated with ALK degrader TL13-112 for 8h. n=3 technical replicates. Data are shown as mean ± s.d. **P<0.01, ****P<0.0001. Significance was determined by unpaired, two tailed Student’s t-test.

We then tested whether NPM-ALK kinase activity directly controlled CD45 expression as we reported for other TCR-related molecules in ALK+ ALCL (6). We treated 3 ALK+ ALCL cell lines (TS, SU-DHL1 and JB6) with sublethal concentrations of crizotinib (from 10nM to 40nM) in order to inactivate NPM-ALK without affecting cell viability (**Figure 1C**). We observed that prolonged treatment with crizotinib (up to 96h) induced CD45RO upregulation in all cell lines in a dose-dependent manner (**Figure 1D**). We concluded that ALK kinase activity controlled CD45 protein expression in ALCL cells. When NPM-ALK was either silenced with an inducible shRNA system or degraded by TL13-112, a newly developed PROTAC ALK degrader (**Figure 2A** and **Supplementary Figure 1A**), a two-to-three-fold increase of CD45 surface expression was observed (**Figure 2B** and **Supplementary Figure 2**). Specifically, the CD45RO isoform was affected by NPM-ALK, whereas the CD45RA isoform was not, as observed by flow cytometry and western blot analyses (**Figure 2B** and **Supplementary Figures 1A, B**). We and others previously reported that ALK oncogenic activity controls protein expression through transcriptional regulation or epigenetic silencing (6, 25, 26). We then investigated the mechanisms of ALK regulation on CD45 expression in ALCL cell lines. We found that degradation of ALK by TL13-112 induced a significant increase in CD45 mRNA levels, and consistently with protein data, only the CD45RO isoform was affected by NPM-ALK (**Figure 2C**). We observed the same CD45 mRNA upregulation when NPM-ALK was knocked down by a specific ALK shRNA (**Supplementary Figure 1C**). Overall, our results suggest that ALK regulates CD45 by repressing its transcription.

### Oncogenic NPM-ALK activity promotes CD45 downregulation *via* STAT3 in ALK+ ALCL

**Figure 3:**
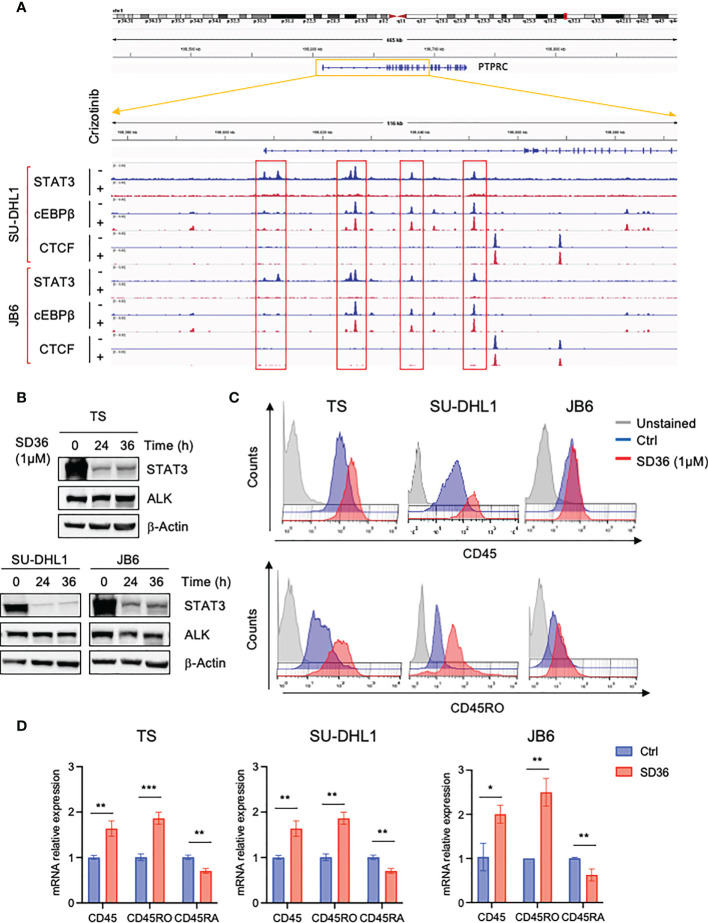
CD45 and CD45RO are derepressed by ALK *via* STAT3. **(A)** STAT3, C/EBP-β and CTCF ChIP-seq tracks at CCR7 gene on two representative ALK+ ALCL human cell lines (JB6 and SU-DHL1) treated with crizotinib (300 nM) for 3h. Experiment was performed one time on two independent cell lines with similar results. **(B)** Immunoblot analysis performed on three ALK+ ALCL cells (TS, SU-DHL1 and JB6) treated with STAT3 degrader SD36 (1μM) for the indicated time-points. Total cell lysates were blotted with the indicated antibodies. β-actin was used as a loading control. A representative Western blot out of two is shown for each cell line. **(C)** CD45 and CD45RO cell surface expression intensity in ALK+ ALCL cell lines (TS, SU-DHL1 and JB6) treated with STAT3 degrader SD36 (1μM). TS and JB6 cells were treated for 48h and SU-DHL1 for 36h. A representative experiment out of two is shown for each cell line. **(D)** qRT-PCR analysis of CD45, CD45RO, CD45RA mRNA expression performed on ALK+ ALCL cell lines treated with STAT3 degrader SD36 (1μM) for 36h. n=3 technical replicates. Data are shown as mean ± s.d. *P<0.05, **P<0.01, ***P<0.001. Significance was determined by unpaired, two tailed Student’s t-test.

Since we previously demonstrated that the transcription factor STAT3 is a critical downstream effector of oncogenic NPM-ALK, we explored the involvement of STAT3 in CD45 transcriptional repression. To investigate whether STAT3 might directly regulate the expression of CD45, we analyzed ChIP-seq data of ALK+ ALCL cells that we previously generated by treating ALK+ ALCL cell lines with crizotinib for 3 hours (27). We found four STAT3 binding sites in the promoter of the *PTPRC* gene that encodes CD45 and observed that STAT3 binding was strongly reduced upon ALK inhibition (**Figure 3A**). We also looked at C/EBP-β, another key downstream effector of ALK in ALCL (28) and found that C/EBP-β bound to the *PTPRC* gene at the same sites of STAT3, but its binding was not affected by ALK inhibition. These findings prompted us to study further the role of STAT3 in CD45 (PTPRC) regulation in ALK+ ALCL and excluded a role for C/EBP-β in ALK-mediated repression of CD45. We took advantage of a new class of compounds, named PROTAC degraders, recently developed to degrade STAT3 (29). Degradation of STAT3 by the degrader SD36 was specific and did not affect ALK expression (**Figure 3B**) and caused increased CD45 expression at protein and mRNA levels (**Figures 3C, D** and **Supplementary Figure 3**). In line with ALK degradation, only the CD45RO isoform was mainly affected by STAT3 degradation, however a significant slight decrease of the CD45RA isoform was also observed at mRNA level (**Figure 3D**). These results show that ALK regulates CD45 expression *via* STAT3.

### CD45 contributes to crizotinb resistance in ALK+ ALCL

**Figure 4:**
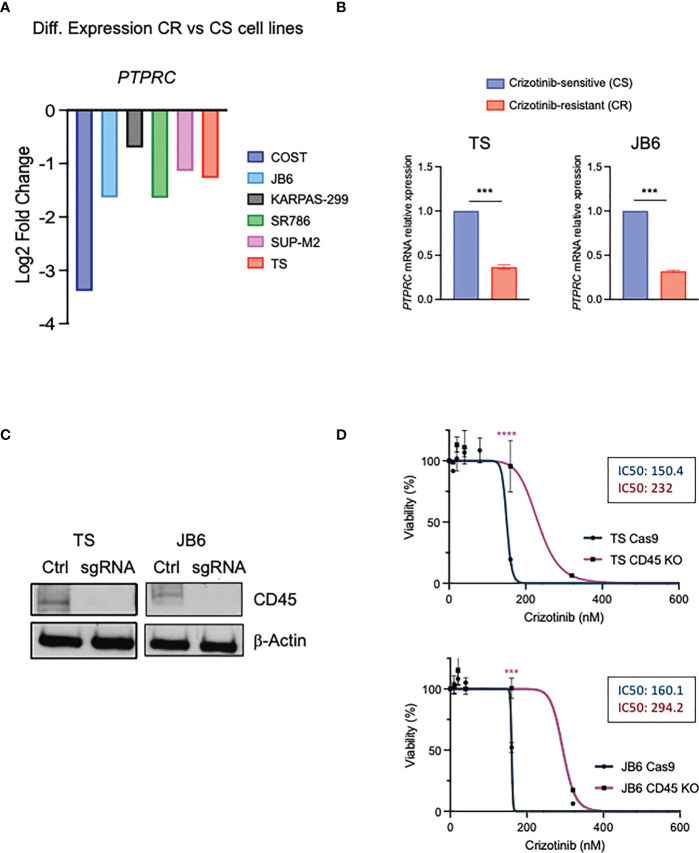
CD45 contributes to crizotinib resistance in ALCL. **(A)** Differential expression levels of *PTPRC* gene based on the RNA-seq data in paired crizotinib-sensitive (CS) and crizotinib-resistant (CR) ALK+ ALCL cells. **(B)** qRT-PCR analysis of PTPRC (CD45) mRNA expression performed on crizotinib-sensitive (CS) and crizotinib- resistant (CR) ALK+ ALCL cell lines (TS and JB6). *PTPRC* mRNA relative expression to paired CS cells is shown. Data are shown mean ± s.d., n=3 technical replicates. **(C)** Immunoblot analysis of TS and JB6 infected with a sgRNA targeting ex7 of *PTPRC* gene (see Materials and Methods) to obtain CD45 knock out cells. Cell lysates were blotted with the indicated antibodies. β-actin was used as a loading control. **(D)** Dose-response curves of ALK+ ALCL cell lines CD45 knock out (TS and JB6) treated with increasing concentrations of crizotinib for 72 h. Data are shown mean ± s.d., n=3 technical replicates. ***P<0.001, ***P<0.0001. Significance was determined by unpaired, two tailed Student’s t-test.

Using a genome-wide loss-of-function screen in ALK+ ALCL cells, we previously found that the non-receptor tyrosine phosphatases PTPN1 and PTPN2 were associated with resistance to ALK TKI (30). In the same data set we also detected *PTPRC* (CD45) among genes whose loss could contribute to crizotinib resistance (30). In parallel, we performed an RNA-sequencing analysis on crizotinb-resistant lymphoma cell lines generated *in vitro* by growing cells in increasing concentrations of crizotinb for 6 months (manuscript under revision). Interestingly, the *PTPRC* gene was differentially expressed between parental crizotinib-sensitive and crizotinib-resistant cells, and when we narrowed our analysis to phosphatases (both receptor and non-receptor phosphatases), *PTPRC* was significantly downregulated in 6 crizotinib-resistant cells out of 7 (**Figure 4A**). We further confirmed *PTPRC* mRNA reduction in resistant cells by quantitative PCR in TS and JB6 cell lines that also showed wild-type NPM-ALK (**Figure 4B**). To investigate the role of CD45 loss or downregulation in resistance to ALK TKIs, we deleted CD45 by CRISPR/Cas9 system using a sgRNA specific for CD45 in 2 different ALK+ ALCL cell lines (TS and JB6) (**Figure 4C**). We then tested sensitivity to crizotinib by treating CD45 KO cells with increasing concentrations of crizotinb and observed that CD45 KO cells were less sensitive to crizotinb than CD45 WT cells (**Figure 4D**). Altogether, we conclude that CD45 is involved in crizotinib-resistance and that its loss could contribute to resistance to TKI treatment in ALCL.

## Discussion

In this study, we found that NPM-ALK oncogenic activity regulates the transmembrane tyrosine phosphatase CD45 in ALK+ ALCL. We show that NPM-ALK downregulates CD45 through the master regulator of ALK-oncogenic signaling STAT3. CD45 is fundamental for proper antigen receptor signaling in T and B cells. In T lymphocytes, CD45 acts as a rheostat of TCR signaling by positively and negatively modulating the activity of the SRC-family kinase protein LCK (31). Here, in ALK+ ALCL the repression of CD45 is consistent with the repression of proximal-TCR signaling pathway by NPM-ALK, as we previously reported in ALK+ ALCL (6). Pawlicki et al. have recently demonstrated that the reprogramming induced by NPM-ALK is central for T cell transformation and full acquisition of a neoplastic phenotype (26). However, in contrast to other TCR related molecules, such as CD3, LCK or ZAP70, CD45 is only downregulated and not completely repressed because altered levels of CD45 can affect T cell development within the thymus and T cell survival in the periphery (32–34). Nonetheless, the regulation of the TCR signaling by CD45 is likely less fundamental in ALK+ ALCL for two main reasons: i) the lack of LCK expression, the major substrate of CD45; ii) the control of the TCR pathway by oncogenic NPM-ALK that represses proximal TCR molecules and activates distal TCR related molecules (6). We also demonstrated that ALK+ ALCL cells mainly express the CD45RO isoform, showing a CD45RO^high^/CD45RA^low^ phenotype. Interestingly, the expression of this isoform was dependent on NPM-ALK activity, whereas the isoform CD45RA was not affected when NPM-ALK was inhibited by ALK TKIs or degraded by ALK PROTAC. CD45RO is mainly expressed in activated or memory T cells and is less responsive to TCR ligation than CD45RA or other CD45 isoforms (12). Indeed, in T-lymphocytes splicing from CD45RA to CD45RO seems to function as a feedback mechanism to switch off prolonged TCR signaling that can have a negative effect on T cell survival (12). In addition, ALK+ ALCLs need to downregulate TCR signaling because full TCR signaling is not compatible with the fitness of lymphoma cells (35). Therefore, we can hypothesize that downregulation of CD45 is needed to downregulate TCR signaling in ALK+ ALCL to protect lymphoma cells from apoptosis induced by TCR over-signaling (35). Altogether, CD45 downregulation and likely the preferential expression of CD45RO isoform could provide a selective advantage for transformed T cells. This hypothesis implies that normal levels of CD45 could generate an excess of oncogenic signaling decreasing lymphoma cells fitness.

In ALK+ ALCL CD45 can be also related to other signaling pathways, such as the JAK/STAT pathway. In hematopoietic cells, CD45 inhibits cytokine receptor signaling by negatively regulating JAK kinases, and therefore controlling cell proliferation in response to cytokines (15). Recently, Porcu et al. reported *PTPRC* nonsense or missense mutations that led to CD45 inactivation or loss in T-ALL. Moreover, in these tumors *PTPRC* mutations usually occurred with activating mutations in pathways negatively regulated by CD45, such as JAK1 or LCK (16). In these T-ALL cases, the downregulation of CD45 was associated with increasing JAK/STAT signaling and interestingly correlated with sensitivity to JAK inhibitors (17). Thereby, CD45 can act as a tumor suppressor by directly dephosphorylating the JAK tyrosine kinases in response to cytokine signaling. To support a role as a tumor suppressor for CD45, loss of function genetic alterations of *PTPRC* gene have been recently reported in peripheral T cell lymphoma, not otherwise specified (PTCL, NOS) (36). Because of the absence of the major substrate of CD45, LCK, it is plausible that in ALK+ ALCL the CD45 phosphatase activity affects the JAK/STAT pathway therefore playing a role as a tumor suppressor. We can speculate that downregulation of CD45 by oncogenic NPM-ALK is fundamental to keep an active JAK/STAT pathway to sustain lymphoma cell survival. STAT3 dephosphorylation could be detrimental to lymphoma cells since we and other demonstrated that silencing STAT3 using specific shRNAs or degrading STAT3 with the PROTAC degraders, a new class of compounds, induce lymphoma cell-cycle arrest and and/or apoptosis (29). In addition, STAT3 can drive resistance to crizotinib as a bypass mechanism to ALK inhibition in ALCL (37). Remarkably, CD45 is repressed through STAT3 in a sort of autoregulatory loop. As demonstrated by ChIP-seq, STAT3 can specifically recognize sequences on the promoter of *PTPRC* gene therefore modulating its expression (**Figure 3A**). However, this latter hypothesis needs further investigation.

Remarkably, phosphatases can influence the response to TKI treatment as reported for PTPN1 (PTP1B) (38), PTPN6 (SHP1) (39) and PTPRC (CD45) (40) for BCR-ABL TKIs treatment in chronic myeloid leukemia or for PTPN11 (SHP2) for acquired resistance to ALK-TKIs in ALK-rearranged non-small cell lung cancer (NSCLC) (41). We also recently reported that the phosphatases PTPN1 and PTPN2 contribute to crizotinib-resistance in ALK+ ALCL by regulating NPM-ALK and SHP2 phosphorylation (30). These studies proved that phosphatases could represent actionable targets to treat different tumors and bypass or even prevent acquired resistance to TKIs. For example, SHP2 inhibition is currently under investigation in several clinical trials for advanced solid tumors (https://clinicaltrials.gov/ct2/results?cond=&term=shp2&cntry=&state=&city=&dist=).

Here, we show that CD45 was associated with ALK resistance to crizotinib in ALCL. We identified the loss or the downregulation of the *PTPRC* gene in resistant ALCL cells by two different approaches: i) an RNA-sequence analysis in crizotinb-resistant cells generated *in vitro* by exposing cells to increasing concentration of crizotinib, and ii) a genome-wide CRISPR/Cas9 loss-of-function screen in ALK+ ALCL cells under crizotinib treatment (30). From both approaches non-receptor and receptor tyrosine phosphatases emerged as mediators of TKI resistance in ALCL (30). Besides *PTPRC*, we found that *PTPN11* (SHP2) was upregulated in resistant cells (**Figure 4A**) accordingly with our previous study (30). By this approach, we found that other tyrosine PTPs were downregulated or upregulated in resistant cells (**Figure 4A**), but further investigation is necessary to clarify their contribution to ALK TKI resistance. When we knocked out CD45 by the CRISPR/Cas9 approach ALCL cells were less sensitive to crizotinib treatment. In cells that do not express CD45, cell proliferation and survival are increased through the lack of inhibition of the JAK/STAT pathway (15, 16). Therefore, in crizotinib-resistant ALCL cells the downregulation of CD45 is probably correlated to the loss of negative regulation on the antiapoptotic JAK/STAT pathway. Similarly, Greenplate et al. reported downregulation of CD45, at relapse, in a case of T-cell prolymphocytic leukemia (P-TLL) resistant to ruxolitinib treatment. Interestingly, in this patient downregulation of CD45 correlated with a decrease in the levels of phosphorylated STAT5 (42). However, the impact of CD45 on treatment response to inhibitors or chemotherapy is still contradictory. For example, in CML using a CRISPR/Cas9-mediated knockout approach, the loss of *PTPRC* significantly improved response to both imatinib and nilotinib, first and second-generation BCR-ABL TKIs, respectively (40). Consistently, previous works identified *PTPRC* (CD45) as a potential prognostic marker since *PTPRC* higher expression correlated with a poor prognosis in precursor B-cell and T-cell acute lymphoblastic leukemia (43, 44).

In conclusion, we explored the role of CD45 in ALK+ ALCL and demonstrated that CD45 downregulation depends by NPM-ALK activity in ALK+ ALCL and that its depletion leads to ALK-TKI resistance. Further investigation could explore the mechanisms influenced by CD45 downregulation in ALK+ ALCL and eventually find new actionable targets to bypass resistance.

## Data availability statement

The datasets presented in this study can be found in the NCBI Gene Expression Omnibus (GEO). Accession number: GSE173306, Token: ohqdkaawnfkpbon. Further inquiries can be directed to the corresponding author/s.

## Author contributions

GM, EK, MM, CM, CA GG, RC and CV performed and analyzed experiments. MA performed RNA sequencing experiments on crizotinib-sensitive and resistant ALCL cell lines. MO, RCa, analyzed RNA sequencing data of crizotinib-sensitive and resistant ALCL cell lines. CV and RC conceived, supervised the project and wrote the manuscript. All authors contributed to the article and approved the submitted version.
