# Supplementary material for: Regulation of CD45 phosphatase by oncogenic ALK in anaplastic large cell lymphoma
Source: Front Oncol. 2023 Jan 9;12:1085672. doi: 10.3389/fonc.2022.1085672 (PMC9869957; doi:10.3389/fonc.2022.1085672)
Supplement: Supplementary file 1 [file DataSheet_1.pdf]

Mura et al. - Supplementary Figures

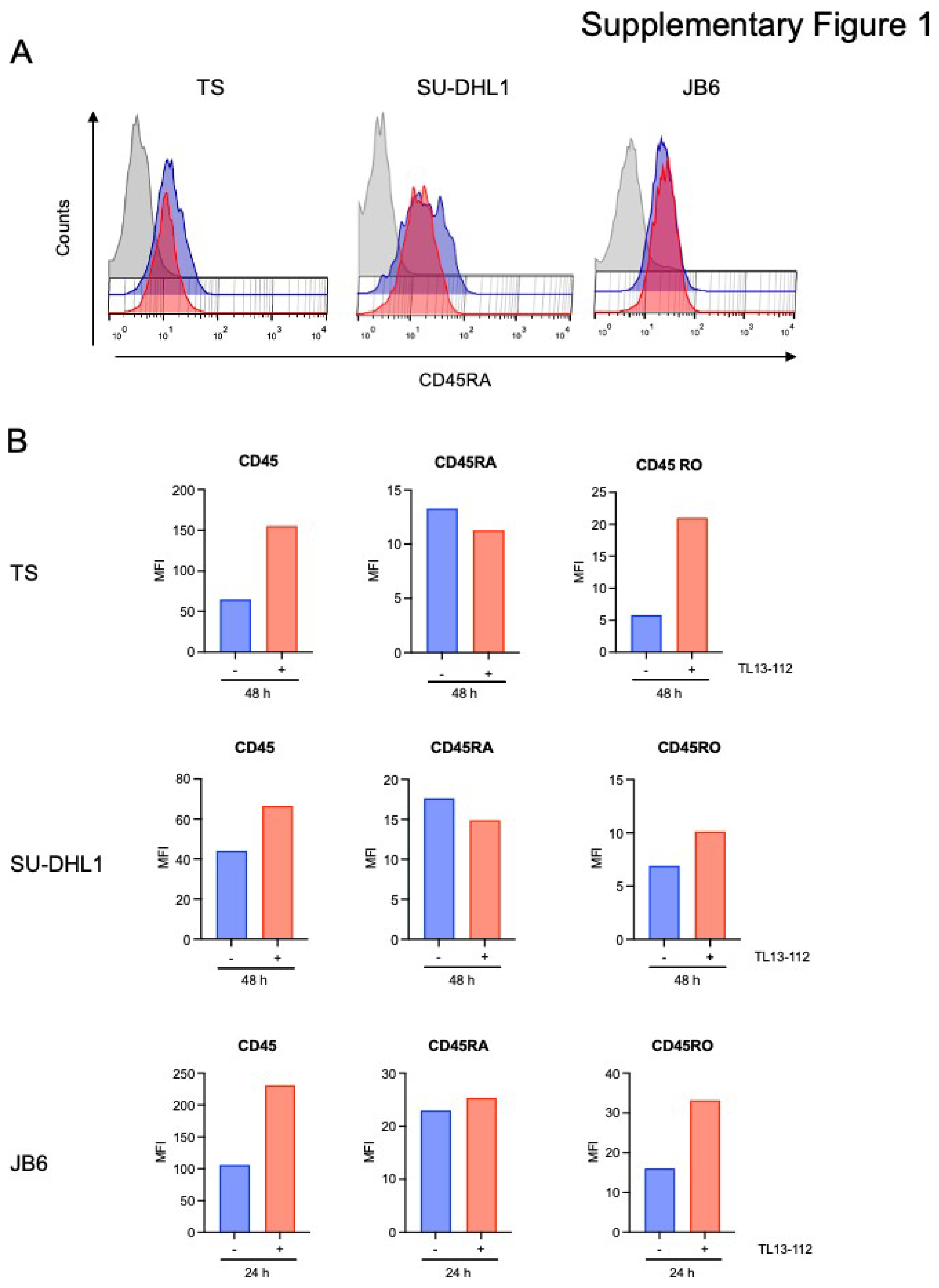

Supplementary Figure 1.

A) CD45RA cell surface expression intensity measured by flow-cytometry in ALK+ ALCL cell lines treated with ALK degrader TL13-112. TS cells were treated using a concentration of 25nM for 48h, SU-DHL1 using a concentration of 10nM for 48h and JB6 using a concentration of 50nM for 24h. B) Histograms show MFI of CD45, CD45RA, CD45RO on cell surface expression in ALK+ ALCL cells treated with the indicated concentration of TL13-112 and at the indicated time. A representative experiment out of two is shown for each cell line for A and B.

Supplementary Figure 2

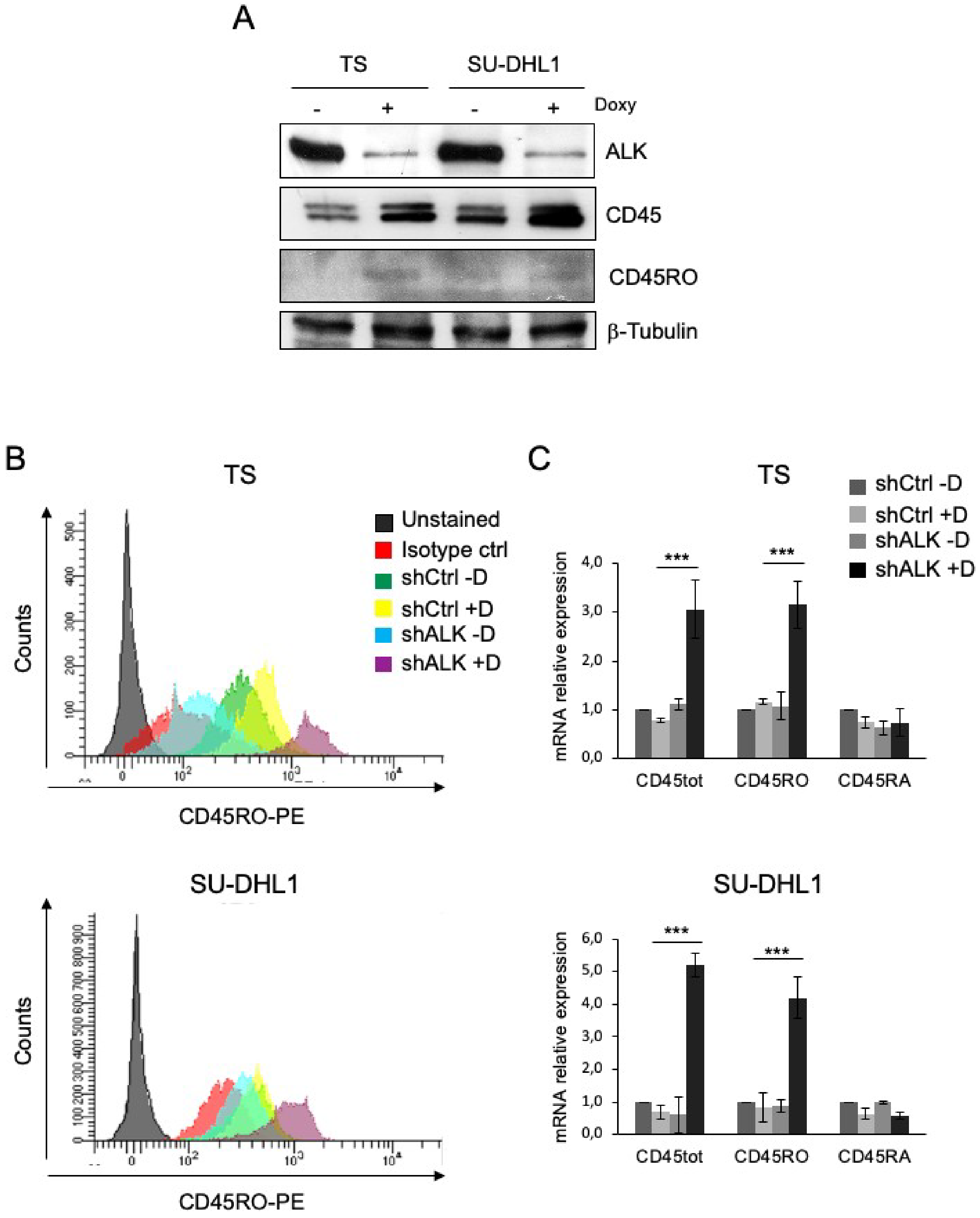

Supplementary Figure 2.

A) Immunoblot Analysis performed on TS-TTA-A5 and SU-DHL 1-TTA-A5 (TS shALK and SU-DHL1 shALK) induced with doxycycline (1μg/ml) for 24h. Cell lysates were blotted with the indicated antibodies. β-tubulin was used as a loading control. B) Two ALK+ ALCL cell lines (TS and SU-DHL1) transduced with pLVTH-ALK-A5/GFP (shALK) and with the mutated pLVTH-ALK-A5M/GFP (shCtrl) were cultured with doxycycline (doxy 1 μg/ml) for 96h and subsequently analyzed by flow cytometry to analyze CD45RO cell surface expression. The experiment and Western blot were performed one time on two independent cell lines with similar results. C) shCtrl or shALK-transduced TS-TTA and SU-DHL1-TTA cells were treated with doxy for 96 hours, and mRNA expression for CD45, CD45RO and CD45RA was determined by qRT-PCR. n=3 technical replicates. Data are shown as mean ±s.d. ***$P<0.001$. Significance was determined by unpaired, two tailed Student's t-test.

Supplementary Figure 3

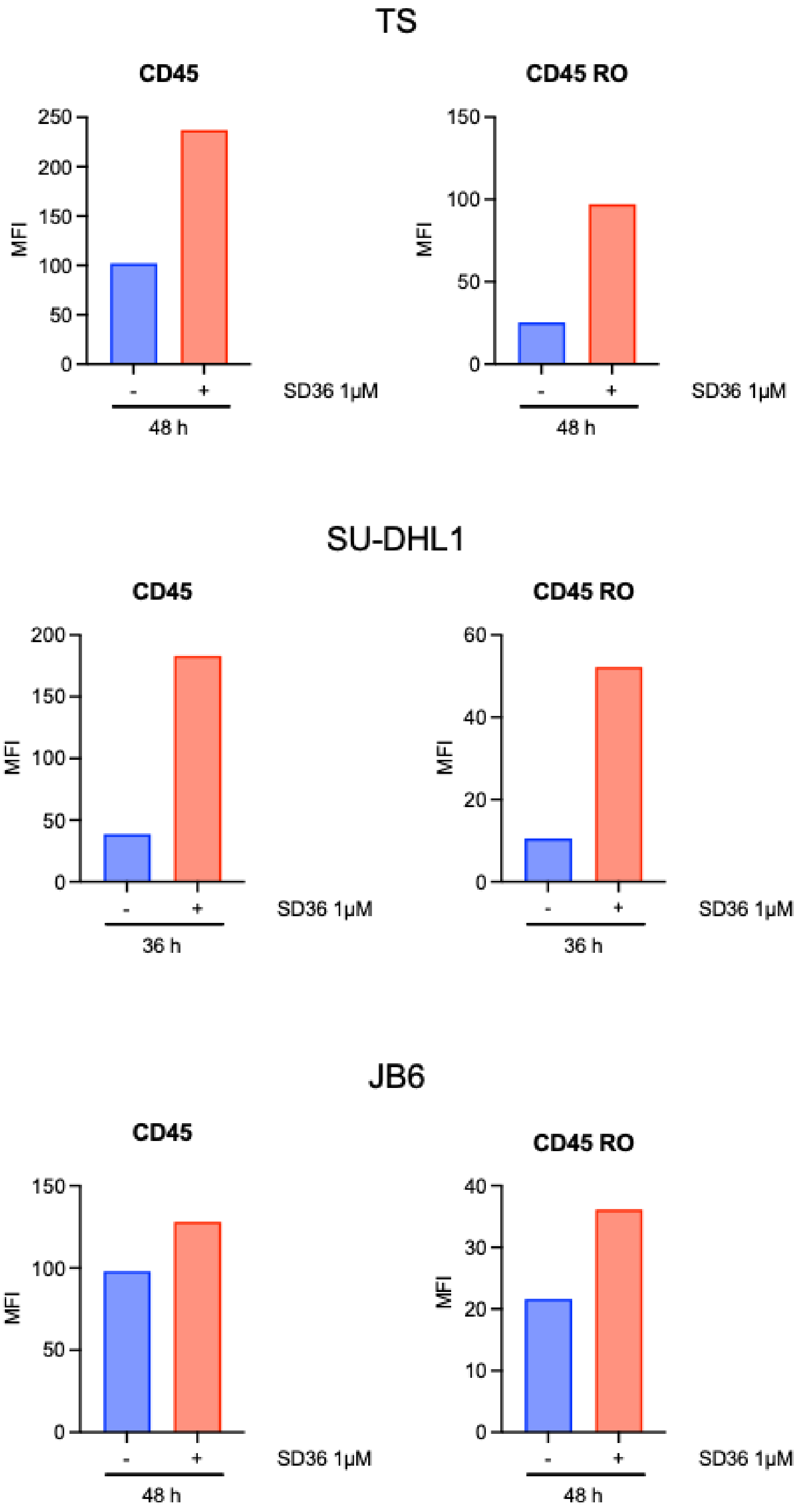

Supplementary Figure 3.

Histograms show MFI of CD45 and CD45RO on cell surface expression in TS, SU-DHL and JB6 (ALCL ALK+ cell lines) treated with SD36 1µM at the indicated time. A representative experiment out of two is shown for each cell line.
